# The long non‐coding RNA OLC8 enhances gastric cancer by interaction with *IL‐11*

**DOI:** 10.1002/jcla.22962

**Published:** 2019-07-05

**Authors:** Rongjia Zhou, Zhanbin Wu, Xixiang Deng, Haojun Chen

**Affiliations:** ^1^ Department of Gastroenterology Panyu Central Hospital Guangzhou Guangdong China

**Keywords:** gastric cancer, IL‐11, lncRNA, OLC8

## Abstract

**Background:**

The gastric cancer (GC) represents a common malignancy especially in China. Long non‐coding RNAs (lncRNAs) are critically involved in various types of cancer. However, the underlying mechanisms of OLC8 in gastric cancer are still largely unknown.

**Methods:**

The lncRNA profiling was used to identify novel lncRNAs associated with GC. The expression of OLC8 was quantified using qRT‐PCR. Migration and viability assays were performed to evaluate the in vitro effects. Xenograft tumor models were conducted to investigate the in vivo oncogenic potential. RNA‐seq was used to identify *IL‐11* as OLC8 binding partner.

**Results:**

In current study, we have identified a novel lncRNA termed OLC8. OLC8 was significantly overexpressed in gastric cancer specimens and cell lines. In vitro experiments showed that OLC8 facilitated migration and viability of MKN1 and AGS cells. As expected, in vivo experiments also confirmed an oncogenic role for OLC8. Mechanistic study indicated that OLC8 associated with *IL‐11* transcripts. The OLC8‐*IL‐11* binding greatly impaired the degradation of *IL‐11* mRNAs. Not surprisingly, enhanced expression of IL‐11 could increase STAT3 activation to favor gastric cancer development.

**Conclusions:**

Our current research has identified OLC8 as a novel oncogenic lncRNA in IL‐11/STAT3 signaling, and OLC8 may constitute a potential target for gastric cancer intervention.

## 1 INTRODUCTION

The gastric cancer (GC) belongs to one of the most malignant tumors worldwide with fairly high mortality rates.^1^ The gastric cancer owns a significantly high incidence rate especially in China.^2^ Unfortunately, it is rather difficult for early diagnosis for gastric cancer and this usually leads to the situation with diagnosis at advanced stages. Therefore, the 5‐year survival rates are poor among gastric cancer patients.^3^ Chemotherapy and radiotherapy are the main strategies for gastric cancer treatment, whereas toxicity or drug resistance has formed remarkable obstacles.^4^ Therefore, enhancing our understanding about gastric cancer pathogenesis may help develop novel approaches for gastric cancer treatment.

Extensive investigations have focused on regulatory roles of protein‐coding genes.^5^ However, the human genome is pervaded with non‐coding sequences. The long non‐coding RNAs (lncRNAs) are a class of transcripts with more than 200 nucleotides in length with minimal or no protein‐coding ability.^6^ Accumulating evidence has clarified that lncRNAs play essential roles during cancer progression.^7^ Numerous studies have shown that lncRNAs actively participate in a wide range of processes and are frequently dysregulated in various cancers.^8^ For gastric cancer, the oncogenic lncRNA DANCR can target lncRNA‐LET to advance migration and metastasis.^9^ Another report showed that lncRNA ZEB2‐AS1 upregulates ZEB2 to activate Wnt/β‐catenin signaling and therefore positively correlates with gastric cancer tumorigenesis.^10^ lncRNA‐MALAT1 can increase the density of vasculogenic mimicry and upregulate MMP‐2, MMP‐9, and β‐catenin to facilitate gastric cancer metastasis.^11^ Although there is an ever‐increasing knowledge on the role of lncRNAs in gastric cancer, our understanding is still limited due to a large repository of unknown ones.

In current study, using profiling‐based methods, we have identified a novel intergenic lncRNA AC104986.2 (ENSG00000253948.1), which we named OLC8 (oncogenic long non‐coding RNA on chromosome 8) and is critically involved in gastric cancer progression. We noted that OLC8 is frequently upregulated in gastric cancer tissues or cell lines compared with normal ones. OLC8 displays multiple in vitro effects by promoting viability and migration of gastric cancer cells. Furthermore, OLC8 also enhances xenograft tumor growth in vivo. Mechanistic study by RIP‐seq argues that OLC8 can interact with and stabilize *IL‐11* mRNA. Decreased degradation of *IL‐11* mRNA may subsequently augment STAT3 signaling to facilitate gastric cancer development. The function of OLC8 is mediated by *IL‐11* as *IL‐11* silence, or an OLC8 mutant deficient in IL‐11 binding failed to magnify the oncogenic effects. The current study uncovers a novel oncogenic lncRNA OLC8 and may provide potential insights into the underlying mechanisms of gastric cancer progression.

## 2 MATERIALS AND METHODS

### 2.1 Cells and reagents

GES‐1, BGC‐823, AGS, MKN1, MGC‐803, and SGC‐7901 cells were cultured in an atmosphere with 5% CO_2_ and in Dulbecco's modified Eagle's medium (DMEM, Sigma) at 37°C with 7% fetal bovine serum and 100 μg/mL streptomycin (Sigma). Normal and gastric cancer cell lines were all obtained from Shanghai Cell Biology Institute. Puromycin was used for lentiviral selection. lncRNA‐OLC8 was first cloned followed by insertion into pWPXL vector (GeneChem) to generate pWPXL‐OLC8 (designated as OLC8). The OLC8 construct with mutations within *IL‐11* mRNA binding sites (OLC8‐Mut) was designed and purchased from GeneChem. An empty pWPXL was used as control (control). The short hairpin RNA (shRNA) targeting OLC8 and a scramble control were both designed by GeneChem. Transfection was fulfilled with Lipofectamine 2000. For details, please refer to Table S1.

### 2.2 Human specimens

Gastric cancer samples were all surgical archives at Guangzhou Panyu Central Hospital from January 2017 to October 2018. Written consent was obtained from all patients. All samples were first treated with liquid nitrogen and then stored at a −80°C refrigerator before experiments. Protocols related to human samples were formally approved by the Human Research Ethics Committee (HREC) at Guangzhou Panyu Central Hospital and in accordance with the 1975 Declaration of Helsinki.

### 2.3 Evaluation of viability

A Cell Counting Kit‐8 Toolkit (CCK‐8, Dojindo) was purchased to quantify the viability following the manufacturer's protocols. After cell culture for 36 hours, cells were re‐suspended and then loaded into a 24‐well plate (1 × 10^5^ cells/well) for 5 days. Notably, 30 μL CCK‐8 solutions were used. The optical density at 450 nm was measured with a SpectraMax M5 microplate monitor (Molecular Devices).

### 2.4 Immunohistochemistry

Immunohistochemistry was performed on deparaffinized sections (5 μm). Sections were hydrated with peroxidase and blocked by 3% H_2_O_2_ for 15 minutes. 50 mmol/L pH 6.5 citrate buffer was used to retrieve antigens for 20 minutes. TBS with Tween‐20 was used for cooling down specimens twice. Specific primary antibodies were then added and coated for 2 hours followed by being washed with TTBS twice. HRP‐conjugated horseradish secondary antibodies were selected for in situ hybridization (ISH). All sample slides were coated with 3,3′‐diaminobenzidine (No.D8001, Sigma) and visualized by a microscope in our own institution.

### 2.5 Statistics

Statistical analyses were done using SPSS (version 16; SPSS, Inc). At least triplicates were performed for all experiments. Data were shown as mean ± SD. Mann‐Whitney test was to determine the statistical significance between two groups, while one‐way ANOVA was performed for multiple groups. *P* < 0.05 was considered significant.

## 3 RESULTS

### 3.1 The OLC8 is a GC‐related lncRNA

**Figure 1:**
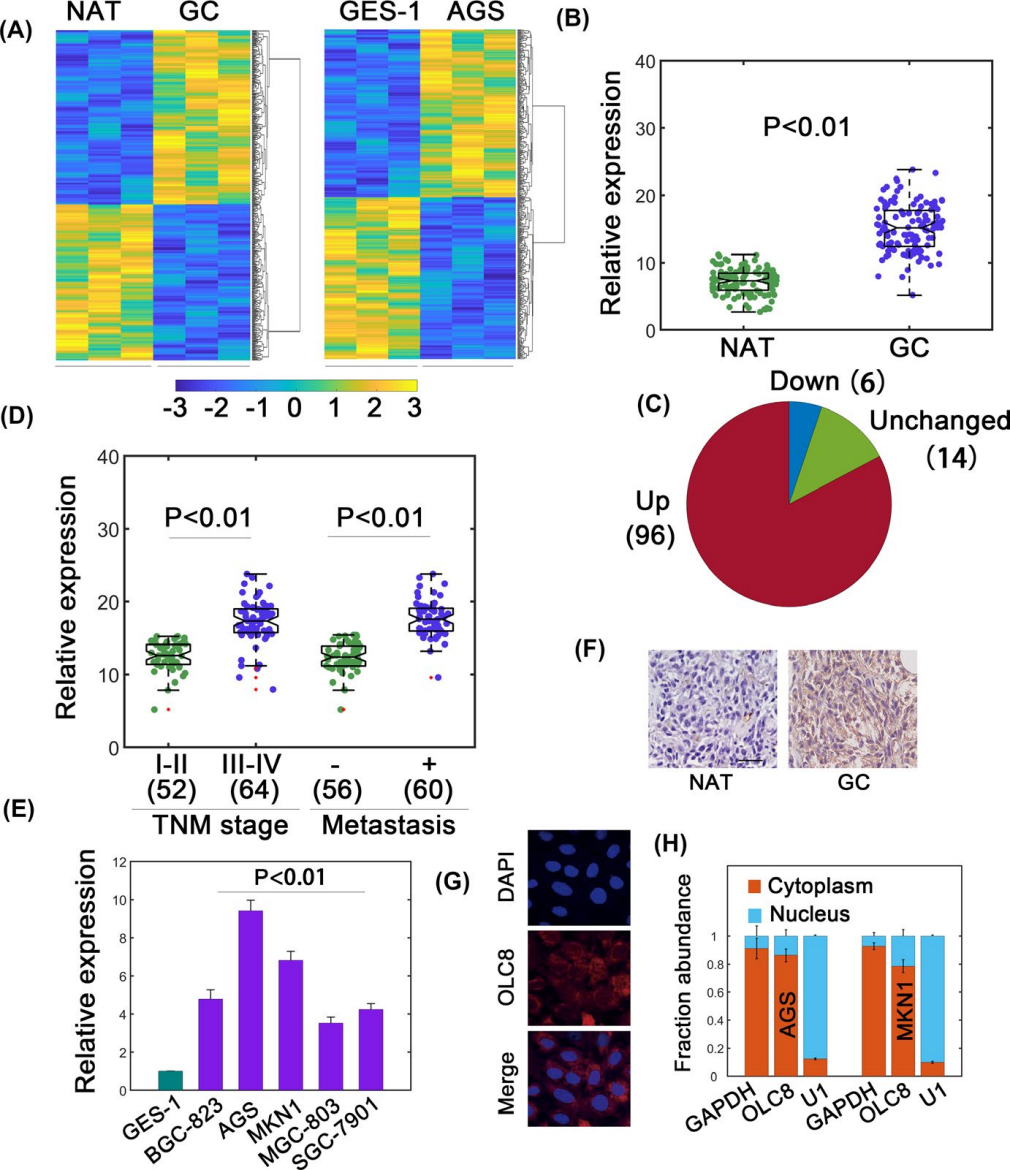
Identification of OLC8 in gastric cancer (GC). (A) lncRNA profiling assays for GC/NAT samples (left) and GES‐1/AGS cells (right). (B) Relative expression (normalized to *GAPDH*) of OLC8 in GC and NATs. Totally, n = 116. (C) Upregulated, unchanged, and downregulated samples were revealed in pie chart. Data were from (B). (D) Relative expression of OLC8 with respect to TNM stages and metastatic status. The number of cases was shown in brackets. (E) Relative levels of OLC8 in GES‐1 and cancerous cell lines. (F) ISH assay demonstrated high OLC8 expression in GC tissues. (G) The localization of OLC8 was revealed by FISH. (H) Nucleocytoplasmic separation assay to quantify the fraction of OLC8 in cytoplasm or nucleus. **P < 0.01

To identify potential GC‐related lncRNAs, we performed lncRNA profiling. GC samples and normal adjacent tissues (NATs) were used (Figure 1A, left). GES‐1 and AGS cells were also subject to profiling (Figure 1A, right). In GC sample/NAT profiling, 214 significantly upregulated lncRNAs were shown (Figure 1A). During GES‐1/AGS profiling, 230 differentially upregulated ones were identified (Figure 1A). By overlapping, we unraveled three novel lncRNAs, which were remarkably increased in both groups (Table S2). As lncRNA AC104986.2 (OLC8) displayed the highest fold induction, we chose it for further analysis. The OLC8 gene is located on chromosome 8 q22.1‐q22.3 (http://www.ensembl.org). It has one annotated transcript in the NCBI database (https://www.ncbi.nlm.nih.gov/) with limited coding potential (score 0.595 compared with 12.246 for *GAPDH*, http://cpc.cbi.pku.edu.cn/). We also used the Coding Potential Assessment Tool (CPAT, http://lilab.research.bcm.edu/cpat/index.php), and the results showed a minimal coding probability 0.0148 in comparison with *GAPDH* (probability ~0.9944). We found that OLC8 was significantly upregulated in GC tissues compared with NATs (Figure 1B). Most samples showed higher OLC8 expression (96/116, Figure 1C). Higher OLC8 levels also positively correlated with advanced TNM stages and metastasis (Figure 1D and Table S3). Meanwhile, OLC8 also associated with tumor size but exhibited no significant correlation with age and gender (Table S3). OLC8 was also upregulated in various GC cell lines (Figure 1E). In situ hybridization further showed higher OLC8 signals in GC specimens (Figure 1F). Furthermore, fluorescence in situ hybridization (FISH) data confirmed a predominantly cytoplasmic distribution for OLC8 (Figure 1G). Subcellular fractionation assay also showed consistent results (Figure 1H). These results identified OLC8 as a candidate oncogenic lncRNA in GC. Since AGS and MKN1 cells showed relatively higher OLC8 expression, we selected these two cell lines for further analysis.

### 3.2 OLC8 promotes GC progression

**Figure 2:**
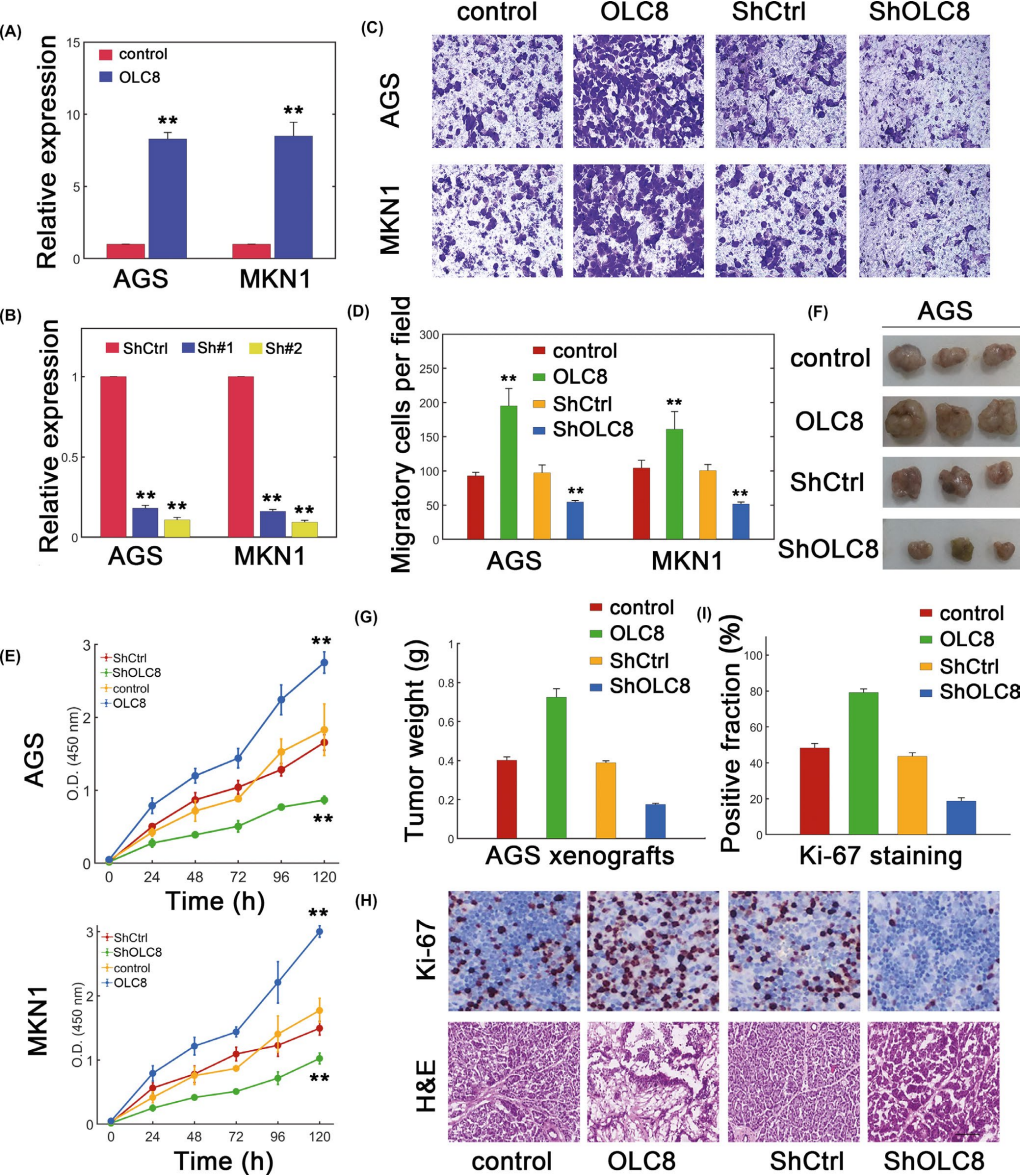
OLC8 facilitates GC progression. (A) Efficiency of lentiviral transfection to overexpress OLC8 in AGS and MKN1 cells. (B) The effect of shRNA‐mediated OLC8 silence in GC cell lines. Sh#1 and Sh#2 represented two designed sequences. Since Sh#2 showed higher efficiency, it was selected as ShOLC8. (C) Migration assay for AGS and MKN1 cells transfected with lentiviral control (control), lentiviral vector containing wild‐type OLC8 (OLC8), scramble control (ShCtrl), or shRNA targeting OLC8 (ShOLC8). (D) Quantification results for (C). (E) Viability assay for AGS (top) and MKN1 (bottom) cells. (F) AGS xenograft tumors with OLC8 knockdown or overexpression. (G) Measurements of data from (F). (G) Ki‐67 and immunohistochemical staining for xenograft tumor slides with altered OLC8 expression. Scale bar: 100 µm. (H) Quantification of Ki‐67 results from (G). **P < 0.01

We next whether OLC8 indeed played a role in GC. We overexpressed or knocked down OLC8 expression, and the efficiencies were verified (Figure 2A and 2B). We then performed migration assay and found that OLC8 overexpression significantly increased the migration in AGS and MKN1 cells (Figure 2C and 2D). Meanwhile, lowering OLC8 levels consistently decreased the migratory capacity (Figure 2C and 2D). We further quantified the viability and demonstrated that overexpressing OLC8 remarkably enhanced the viability, whereas silencing OLC8 attenuated the viability (Figure 2E). We further established in vivo model to investigate the function of OLC8. As expected, AGS cells with OLC8 overexpression dramatically promoted xenograft tumor growth, while decreasing OLC8 expression inhibited tumor growth (Figure 2F and 2G). More positive Ki‐67 staining was observed when OLC8 was overexpressed, whereas depleting OLC8 expression markedly lowered the positive Ki‐67 fraction (Figure 2H and 2I). These results further consolidated that OLC8 may play oncogenic role both in vitro and in vivo.

### 3.3 OLC8 interacts with IL‐11 to modulate the activity of STAT3 signaling

**Figure 3:**
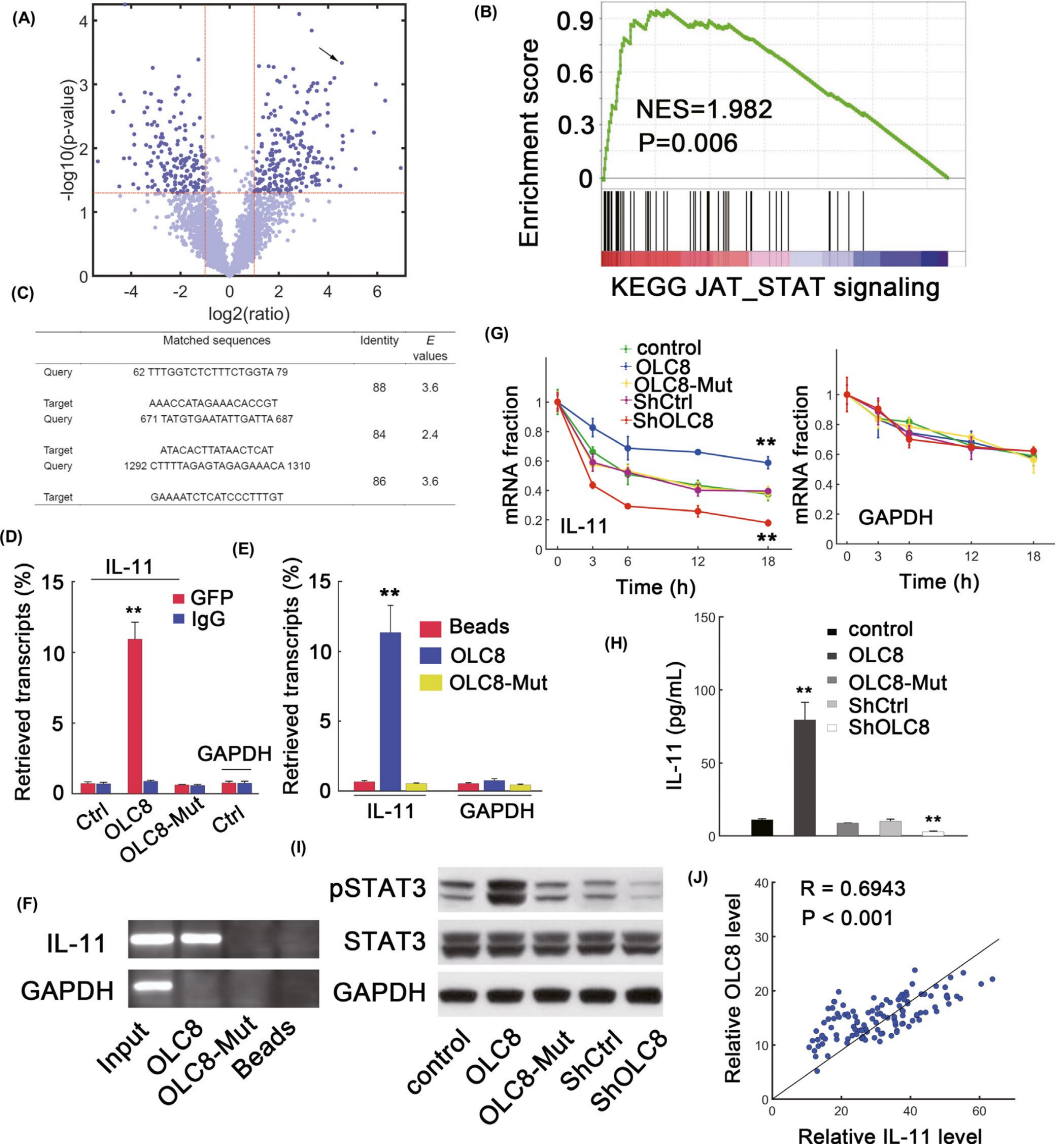
OLC8 interacts with *IL‐11* mRNA to activate STAT3. (A) Comparison between anti‐GFP and non‐specific IgG groups for OLC8 RIP‐derived RNA. The arrow represents *IL‐11*. (B) GSEA for KEGG_JAK_STAT pathway in OLC8 overexpressed AGS cells versus control cells. (C) Predicted interacting domains between OLC8 (query) and *IL‐11* mRNA (target). (D) qRT‐PCR in RIP‐derived RNAs. The fraction of input signals was shown. "OLC8‐Mut" represents the OLC8 construct with mutations in *IL‐11* binding sites. (E‐F) AGS lysates incubated with biotin‐labeled OLC8 (E). The qRT‐PCR assay was conducted after pulling down and extraction of mRNAs (F). (G) The turnover of *IL‐11* and *GAPDH* was depicted through qRT‐PCR. The plot was obtained by normalizing to the values at time 0 after RNA synthesis blockage with α‐amanitin (25 mmol/L) treatment in AGS cells at indicated conditions. (H) IL‐11 concentrations in the culture medium as measured by ELISA in different AGS cells as specified. (I) The levels of phosphorylated STAT3 (pSTAT3) in AGS cells transfected with lentiviral control (control), lentiviral vector containing wild‐type OLC8 (OLC8), lentiviral vector containing mutant OLC8 (OLC8‐Mut), scramble control (ShCtrl), or shRNA targeting OLC8 (ShOLC8). (J) Pearson's correlation between *IL‐11* mRNA and OLC8 in GC samples (n = 116). R denotes the correlation coefficient. **P < 0.01

To explore the mechanisms of OLC8‐mediated GC progression, we applied RNA immunoprecipitation followed by sequencing (RIP‐seq; Figure 3A). The volcano plot was shown (Figure 3A). We found that interleukin‐11 (IL‐11) mRNA was among the highly enriched components (Figure 3A, arrow). Furthermore, GSEA suggested that JAK_STAT pathway from the Molecular Signature Database (MSigDB^12^) was positively enriched in OLC8‐overexpressing cells (Figure 3B). Since IL‐11 can activate STAT3 signaling and promote malignant phenotypes during tumor progression,^13^ we therefore reasoned that *IL‐11* might be the mediator used by OLC8 to regulate STAT3 signaling. We noted that OLC8 overexpression induced higher *IL‐11* mRNA expression and phosphorylated STAT3 accumulation (Figure S1A and S1B). Decreasing OLC8 expression consistently reduced *IL‐11* transcript expression and STAT3 phosphorylation (Figure 1A and 1B). Using BLAST (http://blast.ncbi.nlm.nih.gov/), three complementary regions were found between OLC8 and *IL‐11* mRNA (Figure 3C). Then, all complementary regions were mutated simultaneously to generate OLC8‐Mut. RIP analysis was then performed, and the results showed that OLC8 was highly enriched for *IL‐11* transcripts in comparison with other conditions (Figure 3D). The association between OLC8 and *IL‐11* was also confirmed by pulldowns with biotin‐labeled OLC8 in in vitro experiments (Figure 3E and 3F). To identify whether OLC8 affected *IL‐11* stability, α‐amanitin was added to block the transcription. We therefore observed that OLC8 overexpression substantially decreased the degradation of *IL‐11* mRNA, whereas OLC8‐Mut which was deficient in *IL‐11* binding failed to increase *IL‐11* stability (Figure 3G, left). Notably, *GAPDH* stability was not affected by altering OLC8 levels (Figure 3G, right).

We found that OLC8 overexpression indeed increased the levels of IL‐11 in the supernatants (Figure 3H) as well as STAT3 phosphorylation (Figure 3I). Silencing OLC8 expression reduced IL‐11 secretion and STAT3 activation (Figure 3H and 3I). We found positive correlation between *IL‐11* transcripts and OLC8 levels in selected GC cell lines (Figure S1C). A significantly high correlation between OLC8 and *IL‐11* mRNA was also evident in samples (*R* = 0.6943, *P* < 0.001, Figure 3J). However, introducing OLC8‐Mut failed to elevate IL‐11 levels and STAT3 activation (Figure 3H and 3I). *BCL‐2*, a STAT3 direct transcriptional target,^14^ was upregulated by OLC8 overexpression and reduced via OLC8 knockdown (Figure S1D). OLC8‐Mut again failed to dramatically increase *BCL‐2* induction (Figure S1D). These results suggested that OLC8 could interact with *IL‐11* mRNA to enhance STAT3 signaling.

### 3.4 The effect of OLC8 on gastric cancer requires IL‐11

**Figure 4:**
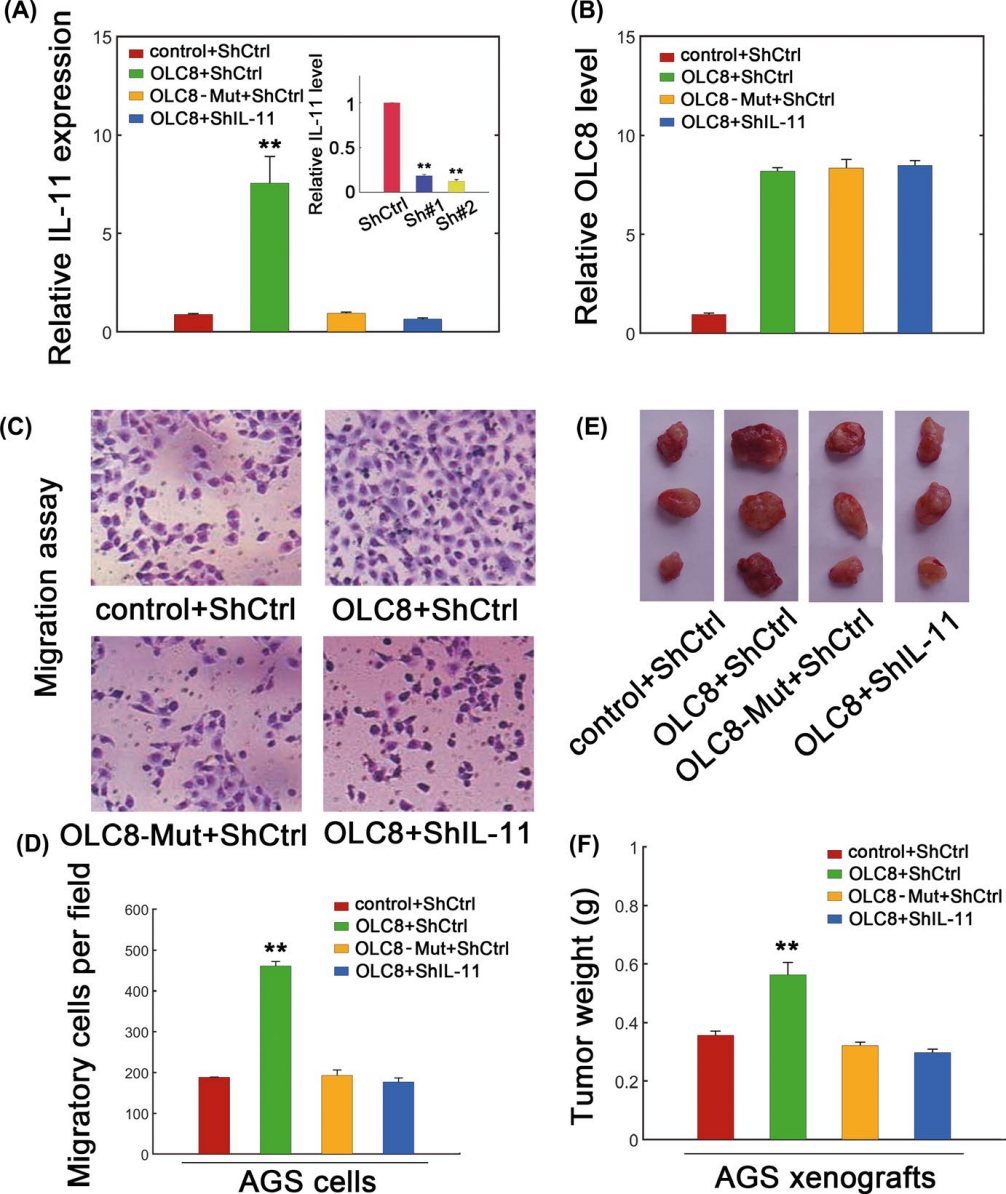
The effect of OLC8 on GC was mediated by IL‐11. (A) Expression of *IL‐11* mRNA in AGS cells transfected with lentiviral control plus scramble shRNA control (control + ShCtrl), lentivirus containing OLC8 plus ShCtrl (OLC8 + ShCtrl), the OLC8 mutant plus ShCtrl (OLC8‐Mut + ShCtrl), or lentivirus containing OLC8 plus ShIL‐11 (OLC8 + ShIL‐11). Efficiency of ShIL‐11 knockdown was displayed as inset. ShIL‐11#2 showed higher efficiency and therefore denoted ShIL‐11. (B) The level of OLC8 in AGS cells. The color labels were the same as (A). (C) Migration assay for different AGS cells as specified. (D) The quantification was shown for (C). (E) AGS xenograft tumors with combinatorial OLC8, OLC8‐Mut, or *IL‐11* transfection as specified. (F) Quantification of (E) as measured via tumor weight. **P < 0.01

We further evaluated whether the effect of OLC8 required IL‐11, and we specifically knocked down *IL‐11* (Figure 4A, inset). Not surprisingly, IL‐11 did not affect OLC8 levels (Figure 4B). However, elevating OLC8 levels dramatically promoted *IL‐11* induction, whereas *IL‐11* silence reversed OLC8‐mediated *IL‐11* enhancement (Figure 4A). Lentiviral transfection containing OLC8 greatly facilitated migration of AGS cells, whereas transfection with the OLC8‐Mut failed to raise the migratory ability (Figure 4C and 4D). As expected, silencing *IL‐11* also obviated the effect of OLC8 overexpression in migration assays (Figure 4C and 4D). To confirm the effect of *IL‐11 *in vivo, we further measured the xenograft tumor growth. Results showed that OLC8 overexpression undoubtedly expedited tumor growth (Figure 4E and 4F). OLC8‐Mut, which failed to bind *IL‐11* mRNA, could not elevate xenograft growth (Figure 4E and 4F). Silencing *IL‐11* induced similar effects irrespective of whether OLC8 was overexpressed or not (Figure 4E and 4F). These data again argued that OLC8 could mediate gastric cancer progression via *IL‐11*.

## 4 DISCUSSION

Accumulating data have led to a conclusion that the lncRNAs actively participate in various biological processes and contribute largely to the tumorigenesis of many cancers.^7^ In current report, we showed that OLC8, which is a novel intergenic lncRNA, can promote gastric cancer development and therefore serves as an oncogenic lncRNA in gastric cancer. Phenotypic studies have demonstrated that OLC8 advances migration and viability in vitro, and the oncogenic role of OLC8 has also been supported in in vivo experiments. OLC8 stabilizes *IL‐11* mRNA to augment STAT3 signaling. As a result, OLC8 may signal through IL‐11/STAT3 pathway to fulfill its oncogenic function.

By RIP‐seq analysis, we have identified OLC8 could bind *IL‐11* mRNA. The interaction between OLC8 and *IL‐11* transcript increased the stability of *IL‐11* mRNA, IL‐11 induction, and activation of STAT3 pathway. Silencing IL‐11 remarkably diminished the oncogenic function of OLC8, suggesting that the role of OLC8 is largely mediated by IL‐11. Notably, IL‐11 belongs to a member of IL‐6 family (eg, IL‐6/‐11/‐27/‐31 and oncostatin M) whose secretion can be induced by myeloid and cancer cells.^15^ Previous data have found that IL‐11 as well as its associated receptor was highly expressed in gastric cancer and significantly correlates with invasion, infiltration, and Lauren's classification.^15^, ^16^ A recent finding suggests that cancer‐associated fibroblasts (CAFs) are highly enriched in samples from gastric cancer patients to facilitate drug resistance largely through enhanced secretion of IL‐11 and activation of IL‐11/gp130/STAT3 pathway.^17^ The raised levels of IL‐11 are primarily through upregulating mucin 1 (MUC1), and interestingly, targeted IL‐11 therapy can reach a promising strategy to cope with gastric cancer via stromal fibroblasts.^18^ Inhibition of IL6/GP130 interaction by bazedoxifene can dramatically repress STAT3 phosphorylation and DNA binding capacity leading to enhanced apoptosis.^19^ IL‐11 overexpression though recombinant *rhIL‐11* introduction facilitates gastric cancer cell metastasis and other phenotypic characteristics.^20^ Consistently, IL‐11 depletion by neutralizing antibodies can also impair colony formation in gastric cancer cells.^20^ All these data argue that IL‐11 undoubtedly serves as a dominant factor which is critically involved in gastric cancer progression. Our current work has shown that OLC8 stabilizes *IL‐11* transcript and increases IL‐11 induction. Manipulating OLC8 might be an effective way to regulate intrinsic *IL‐11* expression. Given the important role of IL‐11 in gastric cancer, our study may provide an alternative route toward IL‐11 regulation and possible disruption of IL‐11/STAT3 pathway.

A variety of studies has converged into the dynamic regulation on STAT3 signaling. For example, lncRNA PVT1 increases angiogenesis of gastric cancer by stimulating STAT3 phosphorylation and VEGFA induction.^21^ lncRNA‐NEAT1 could promote STAT3 expression to reinforce gastric cancer progression, and miR‐506 could neutralize the effect of NEAT1.^22^ Conversely, OLA1P2 can bind STAT3 to suppress its phosphorylation at Tyr705 and formation of homodimers.^23^ Instead, TSLNC8 exerts its anti‐tumor capacity via inhibiting STAT3 phosphorylation at Tyr705 site and this effect is mediated by TSLNC8‐transketolase (TKT) interaction.^24^ We used antibody against Tyr705 to measure the phosphorylation status of STAT3 owing to the fact that the oncogenic effect of Tyr705 has been confirmed in various cancers, while the role of Ser727 phosphorylation on STAT3 is controversial.^25^, ^26^ These positive and negative regulatory effects have substantially complicated the dynamic patterns of gastric cancer progression. Our current research has demonstrated a critical role of OLC8 on the stability of *IL‐11* transcripts upstream in STAT3 signaling pathway and therefore provided additional layer of complexity.

In a recent work, IL‐11/STAT3 might be involved in a positive feedback loop via lncRNA HEGBC.^27^ Notably, positive feedback loops serve as the most essential component during decision‐making processes (eg, biological switch).^28^ Our data have demonstrated that lncRNA OLC8 can directly regulate *IL‐11* stability and may effectively strengthen this positive feedback loop to enhance gastric development. Therefore, OLC8/IL‐11/STAT3 axis may provide a promising target for potential pharmacotherapeutic intervention. However, whether OLC8 could bind other protein factors or acts as a competing endogenous RNA (ceRNA) 29 remains an open question and deserves further investigation.

In summary, we have identified a novel lncRNA termed OLC8, which serves as an oncogenic factor during gastric cancer progression. OLC8 associates with *IL‐11* mRNA and impairs the degradation of *IL‐11* mRNA. Therefore, the novel OL8/IL‐11/STAT3 signaling axis may enrich our understanding of mechanisms of gastric cancer progression.

## CONFLICTS OF INTEREST

The authors declare no competing interests.
