# Supplementary material for: The long non‐coding RNA OLC8 enhances gastric cancer by interaction with IL‐11
Source: J Clin Lab Anal. 2019 Jul 5;33(8):e22962. doi: 10.1002/jcla.22962 (PMC6805327; doi:10.1002/jcla.22962)
Supplement: Supplementary file 1 [file JCLA-33-e22962-s001.docx]

**Online supplementary materials for**

**The long non-coding RNA OLC8 enhances gastric cancer by interaction with *IL-11***

Rongjia Zhou^1†^, Zhanbin Wu^1^, Xixiang Deng^1^, Haojun Chen^1^,

*^1^* *Department of Gastroenterology, Guangzhou Panyu Central Hospital, Guangzhou, 511400, Guangdong, China*

**Supplementary Methods**

**Profiling for lncRNA and RNA-seq**

Total RNAs from samples or cell lines were first extracted by TRIzol RNA Purification Kit (Thermo Fisher Scientific). Then, the samples were subject to sequencing at BGI (Beijing Genomic Institute). Gene expression profiles of the gastric cancer cells transfected with lentiviral control (control) or lentivirus containing OLC8 (OLC8) were also evaluated at BGI with the Hiseq3000 system (Illumina). Genes with fold change (FC)>2 and *P* <0.05 were designated as differentially expressed genes (DEGs).

**RIP-seq**

RIP-seq was done according to previous study [[1](#_ENREF_1)]. Briefly, AGS cells were first transfected with lentiviral vectors for 36 h. Then, RNA immunoprecipitation (RIP) was conducted by Magna RIP™ RNA-Binding Protein Immunoprecipitation Kit (Millipore) together with GFP antibodies following the manufacturer’s guidelines. The sequencing libraries were generated by 200 ng RNAs and TruSeq Stranded Total RNA kits purchased from Illumina. Then, RNA fractions from RIP experiments were determined by NanoDrop ND1000 (Thermo-Fisher Scientific). Differentially expressed transcripts were shown by DEGseq package according to the RPKM values (Fold change, FC>2, False Discovery Rate, FDR < 0.05).

**Nuclear and cytoplasmic fractionation**

AGS and MKN1 cells were resuspended using 2 ml buffer (20 mM HEPES, 1 mM MgCl_2_ and KCl, 1% N-octylglucoside, PH=7.5 RNase and protease inhibitor) for 30 min before homogenization. Cytoplasm fractionates were extracted by centrifugation (2000*g*×20 min). The pellet was treated similar to a previous report [[2](#_ENREF_2)] to deplete minimal cytoplasmic fractionates. RNA extraction kit (Tiangen) was utilized during the experiments. Polyadenylated RNA was then generated by oligo(dT) polystyrene beads (Sigma) to ease purification.

**Reverse transcription quantitative-PCR (RT-qPCR)**

Total RNAs were extracted by TRIzol RNA Purification Kit (Thermo Fisher Scientific) as demonstrated above. A spectrometer was used to determine the concentration. Primers were designed, synthesized and purchased from Sigma (Beijing). The details were listed in Table S1. QuantiTect Reverse Transcription Kit (Qiagen) was used for reverse transcription. Products were diluted to a final concentration of 20 ng/μL and then subject to quantitative PCR. Quantification for expression was fulfilled using real-time PCR in ABI7300 system. Totally, 5 μg cDNA was used as template and *GAPDH* was the control.

**RNA pulldown**

The expression vectors with biotin labels (Biotin RNA Labeling Mix and T7 RNA polymerase, Roche). RNeasy Mini Kit (Qiagen) was used for purification. Notably, 2 mg whole cell lysates (WCL) were incubated with 5 μg of biotinylated transcripts for 2 hours at room temperature. Streptavidin agarose beads (Invitrogen) were used to isolate the complexes. RNAs in pull-downs were then determined by qRT-PCR.

**Fluorescence *in situ* hybridization (FISH)**

The fluorescence-conjugated probes were designed and purchased from by Life Technologies. Non-denaturing conditions were established to treat samples. Then, specific fluorescence-conjugated probes were added for indicated sample slides. Samples were then counter-stained with DAPI and visualized by confocal microscopy in our own institution.

**Gene set enrichment analysis (GSEA)**

The GSEA v2.0 was used to identify the genetic signature in RNA-seq. Statistical significance was determined by comparison between enrichment scores and 2,000 random gene set permutations to obtain the nominal *p* values.

**Western blot**

1×sodium dodecyl sulfate buffer was utilized for preparing protein extracts. Samples (equal quantity of proteins) were separated on 10% SDS-polyacrylamide gels (SDS-PAGE) and transferred onto the nitrocellulose filter membranes (Sigma). The protein samples were then blocked by 4% fat-free milk. Membranes were incubated with specific primary antibodies on a shaker overnight. After washing by Tris-Buffered Saline Tween-20 (TBST) twice, HRP-labeled secondary antibodies were loaded at 20℃ for 1 hour and final blots were visualized with Odyssey infrared scanner. GAPDH was used as the loading control.

**Migration assay**

Cell Migration Assay Kit (ECM510; Millipore) was used to quantify the migration in accordance with the manufacturer’s protocols.

***In vivo* tumorigenesis**

The lentivirus transfected AGS cells were maintained in DMEM for 36 h. Then, 5×10^6^ AGS cells were subcutaneously injected into nude mice (female, BALB/c, 4~5 weeks old). Mice were housed at 20-22℃ environment temperature at strictly controlled 12/12 light/dark cycle with free access to food and water. After 4 weeks, solid tumors were obtained from sacrificed mice and weighed. Slides from tumor samples were covered with 20 nM FISH probes (Life Technologies) for hybridization (15 min) before dehydration. Animal experiments were performed according to the Institutional Animal Care and Use Committee (IACUC) at Guangzhou Panyu Central Hospital.

**Enzyme-Linked Immuno-Sorbent Assay (ELISA) for IL-11**

The IL-11 in culture medium was harvested 48 hours following indicated treatments. Expression was determined by ELISA Human IL-11 ELISA kits (Neobioscience, Shanghai) according to the manufacturer’s protocols.


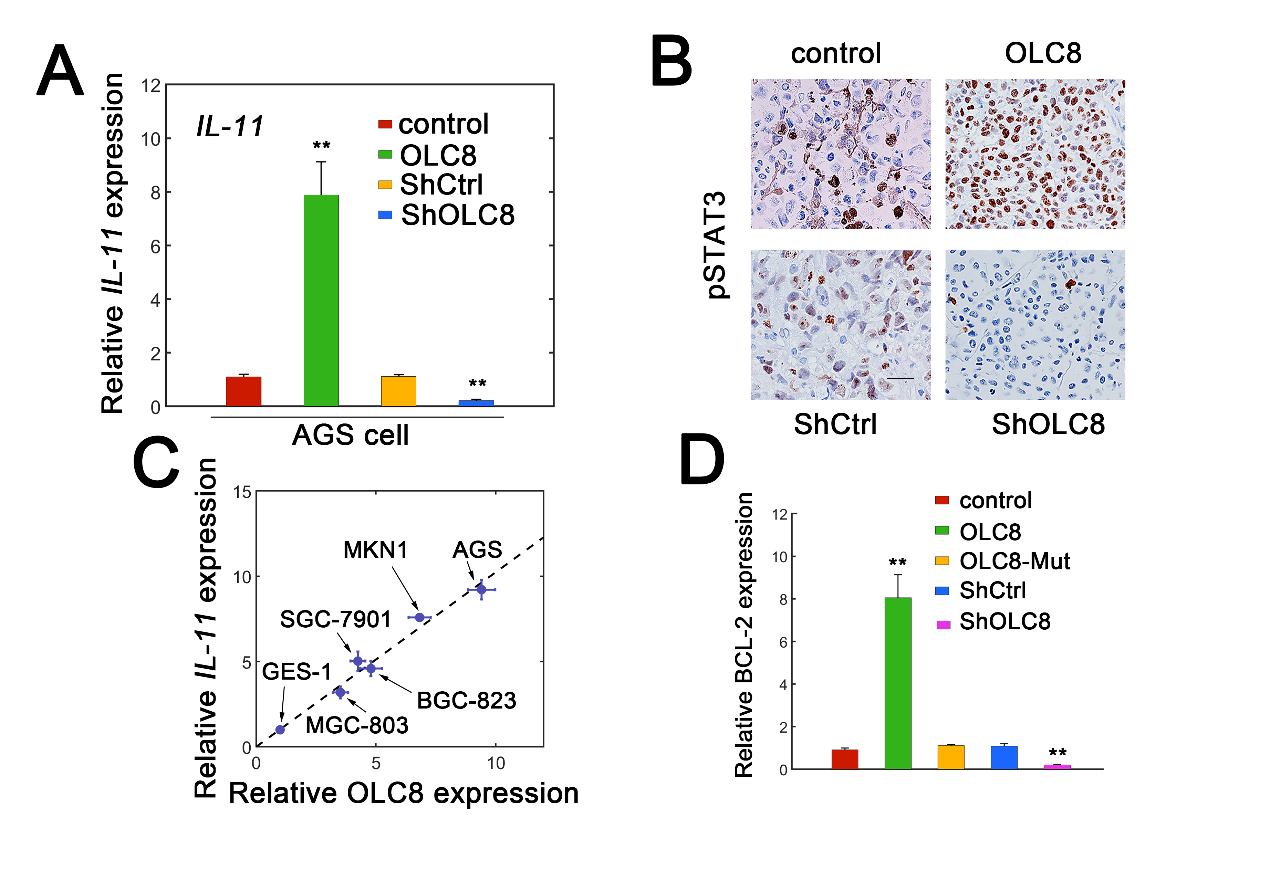


Figure S1. OLC8 activates STAT3 pathway via *IL-11*. (A) Relative expression of *IL-11* mRNA in different AGS cells. (B) Immunohistochemical staining for phosphorylated STAT3 (pSTAT3) by silencing or overexpressing OLC8. Scale bar: 50 µm. (C) Relative *IL-11* mRNA expression in different GC cell lines as specified in Figure 1E. (D) Relative *BCL-2* transcript expression in AGS cells transfected with lentiviral control (control), lentiviral vector containing wild type OLC8 (OLC8), lentiviral vector containing mutant-OLC8 (OLC8-Mut), scramble control (ShCtrl) or ShRNA targeting OLC8 (ShOLC8). **: *P*<0.01.

**Table S1.** Primers and antibodies

| **Name** | **Sequences (Forward/Reverse)** |
| --- | --- |
| OLC8 | GCGTCCATGTCCCAACCTAGTA |
|  | GGGCCATCACTTAACGTG |
| *U1* | CCAGCGATGTTGGCTTCGTTAT |
|  | CGCCACCCTATGTGATGTT |
| *GAPDH* | AGTGTCATGTTCAACCGCAAA |
|  | CACATCTGCTGGAAGGTGGAC |
| *BCL-2* | CACGTTCCGTCTCGACGTGGA |
|  | TAACCGAATAATTACAAGC |
| *IL-11* | GCGGTATGTTGCAGCGAGC |
|  | AGTTCGTATGCAGGAGAGAGC |
| **Primer name** | **Primer sequences** |
| shOLC8 #1 | GAGCTCTTAAATCCGGCTTCGAAGCCGGGGCCATAAGGCTCGTTCACCAATTT |
| shOLC8 #2 | ACTTAGATCGCAGATCAACTGTGGTCAGTAGCAGAATCTCTCGAGGTTATCG |
| ShCtrl | CCGTTCCGAACGTCAACGTTGTCAGACACGTTCGGCGTTTCAGGGGAA |
| shIL-11 #1 | GATCCGTGCACAGCTGAGGGACAAATTTCAAGAGAATTTGTCCCTCAGCTGTGCAC |
| shIL-11 #2 | CAAAAAAGTGCACAGCTGAGGGACAAATTCTCTTGAAATTTGTCCCTCAGCTGTGCAC |
| pWPXL-OLC8-F | CTTAGATTCAACAGACGCCGCAGGCCAGCGT |
| pWPXL-OLC8-R | GCCGAGCTCTTAGGCCTGCTCTGGTTCCTGT |
| pWPXL-OLC8-mut-F | GGGATCCATGTTAGATGATGAAGAGGGACTT |
| pWPXL-OLC8-mut-R | CTGTCTGTATTTGAAGAATACCTTTCGCA |
| **Antibodies/reagents (Catalog Number)** | **Company** |
| STAT3 (#S5933) | Sigma |
| GAPDH（G8795） | Sigma |
| Flag-Tag (#F7425) | Sigma |
| pSTAT3 (Tyr705, SAB4300033) | Sigma |
| HA (#H3663) | Sigma |
| GFP (#G6539) | Sigma |
| α-amanitin (23109-05-9) | Abcam |
| HRP-conjugated secondary antibody  (SAB5300168) | Sigma |
| DAPI (#268298) | Sigma |
| Biotin RNA Labeling Mix (#11685597910) | Roche |
| Ki-67 (#P6834) | Sigma |
| Rabbit IgG Alexa Fluor® 488 Conjugate (#2975) | Cell Signaling |

| Names | Log_2_FC | *p* value | Gene ID |
| --- | --- | --- | --- |
| OLC8 | 7.9892 | 0.0005 | ENSG00000253948.1 |
| RP11-65J3.1 | 5.4561 | 0.0038 | ENSG00000233901.1 |
| RP11-443B7.1 | 6.5724 | 0.0022 | ENSG00000238005.1 |

**Table S2:** Consistently upregulated novel lncRNAs

FC: Fold change.

**Table S3:** Correlation between clinicopathological features and OLC8 expression.

|  |  | OLC8 levels | | *P* value |
| --- | --- | --- | --- | --- |
| Clinicopathological features | NO. | Low  (58) | High  (58) |  |
| **Age** |  |  |  |  |
| < 55 | 55 | 28 (50.9%) | 27 (49.1%) | 0.500 |
| ≥ 55 | 61 | 30 (49.2%) | 31 (50.8%) |  |
| **Gender** |  |  |  |  |
| Female | 54 | 23 (42.6%) | 31 (57.4%) | 0.192 |
| Male | 62 | 35 (56.5%) | 27 (43.5%) |  |
| **TNM stage** |  |  |  |  |
| I-II | 52 | 33 (63.5%) | 19 (36.5%) | 0.007** |
| III-IV | 64 | 25 (39.1%) | 39 (60.9%) |  |
| **Tumor size** |  |  |  |  |
| < 4 cm | 51 | 34 (66.7%) | 17 (33.3%) | 0.003** |
| ≥ 4 cm | 65 | 24 (36.9%) | 41 (63.1%) |  |
| **Metastasis** |  |  |  |  |
| Absent | 56 | 19 (33.9%) | 37 (66.1%) | 0.001** |
| Present | 60 | 39 (65.0%) | 21 (35.0%) |  |

TNM: tumor (T), the extent of spread to the lymph nodes (N), and the presence of metastasis (M) (* P < 0.05, ** *P* <0.01). The median value was used as the cut-off.

**Reference**

[1] J.H. Yuan, F. Yang, F. Wang, J.Z. Ma, Y.J. Guo, Q.F. Tao, F. Liu, W. Pan, T.T. Wang, C.C. Zhou, S.B. Wang, Y.Z. Wang, Y. Yang, N. Yang, W.P. Zhou, G.S. Yang, S.H. Sun, A long noncoding RNA activated by TGF-beta promotes the invasion-metastasis cascade in hepatocellular carcinoma, Cancer Cell, 25 (2014) 666-681.

[2] Z.Z. Chen, L. Huang, Y.H. Wu, W.J. Zhai, P.P. Zhu, Y.F. Gao, LncSox4 promotes the self-renewal of liver tumour-initiating cells through Stat3-mediated Sox4 expression, Nat Commun, 7 (2016) 12598.
